# Streaming algorithms for identification of pathogens and antibiotic resistance potential from real-time MinION^TM^ sequencing

**DOI:** 10.1186/s13742-016-0137-2

**Published:** 2016-07-26

**Authors:** Minh Duc Cao, Devika Ganesamoorthy, Alysha G. Elliott, Huihui Zhang, Matthew A. Cooper, Lachlan J.M. Coin

**Affiliations:** 1 Institute for Molecular Bioscience, The University of Queensland, 306 Carmody Road, St Lucia, Brisbane, QLD 4072 Australia; 2 Department of Genomics of Common Disease, Imperial College London, London, W12 0NN UK

**Keywords:** Nanopore sequencing, Real-time analysis, Pathogen identification, Antibiotic resistance

## Abstract

The recently introduced Oxford Nanopore MinION platform generates DNA sequence data in real-time. This has great potential to shorten the sample-to-results time and is likely to have benefits such as rapid diagnosis of bacterial infection and identification of drug resistance. However, there are few tools available for streaming analysis of real-time sequencing data. Here, we present a framework for streaming analysis of MinION real-time sequence data, together with probabilistic streaming algorithms for species typing, strain typing and antibiotic resistance profile identification. Using four culture isolate samples, as well as a mixed-species sample, we demonstrate that bacterial species and strain information can be obtained within 30 min of sequencing and using about 500 reads, initial drug-resistance profiles within two hours, and complete resistance profiles within 10 h. While strain identification with multi-locus sequence typing required more than 15x coverage to generate confident assignments, our novel gene-presence typing could detect the presence of a known strain with 0.5x coverage. We also show that our pipeline can process over 100 times more data than the current throughput of the MinION on a desktop computer.

**Electronic supplementary material:**

The online version of this article (doi:10.1186/s13742-016-0137-2) contains supplementary material, which is available to authorized users.

## Background

Massively parallel, short-read sequencing has profoundly transformed genomics research [1, 2] and has become the dominant technology for sequencing DNA. However, one inherent limitation of most current technologies is that the sequencing run must finish before data analysis can begin. As a result, sequence analysis algorithms have been designed to make inference on a complete sequencing data set. In contrast, streaming algorithms are applied to a sequence of data events and typically maintain an internal summary of the data, as well as an approximation of the full inference, without needing to store all of the observations [3]. Streaming algorithms have applications in particle and solar physics, computer network analysis and finance [4].

Oxford Nanopore Technologies has recently released a portable MinION sequencing device, which utilises nanopore sequencing technology originally proposed in the 1990s [5]. The key innovation of this device is that it measures changes in electrical current as single-stranded DNA passes through the nanopore and uses the signal to determine the nucleotide sequence of the DNA strand [6, 7]. These sequence data can be retrieved and analysed as they are generated, providing the opportunity to obtain answers in the shortest possible time. Real-time sequencing has many potential applications, especially in time-critical areas such as rapid clinical diagnosis.

In order to realise this potential there is a need to develop streaming bioinformatics algorithms that continually update and report results as each sequence read is generated. To be of practical use – for example to know when to make a diagnosis in the clinic – these algorithms must continuously update not only a point estimate (*e.g.*, which species are present and their proportions), but also confidence intervals in that estimate. Several systems incorporating real-time analysis of MinION data have been developed recently, such as the cloud-based platform Metrichor (Oxford Nanopore), work by Quick et al. [8] and MetaPORE [9], which place the sample on a phylogenetic tree but without estimating the confidence in this assignment.

Here we present a flexible framework for the real-time analysis of MinION sequence data directly as it is sequenced and base-called. The framework can incorporate multiple real-time analyses to suit the problems at hand and can be deployed on a single computer or on a high-performance computing facility and computing cloud. We also present four streaming algorithms for the identification and characterisation of pathogen samples. These algorithms, which are seamlessly integrated into the pipeline, report analysis results along with their confidence levels so that users can decide when to stop a sequencing run.

By sequencing four bacterial isolate samples and a mixture sample on the MinION sequencer, we demonstrate that we can reliably determine the species and strain of a sequenced sample with only 500 reads. This was achieved in less than half an hour of sequencing with the current throughput of the MinION. Furthermore, we show that we can identify most of the drug resistance genes present in a sample within 2 h of sequencing, and the full drug resistance profile within 10 h. The pipeline can perform all these analyses on a single computer at a throughput of over 100 times higher than our best runs. As the throughput of nanopore sequencing is expected to increase, the time to obtain these results will be significantly shortened. Our findings support the potential use of MinION sequencing for the real-time analysis of clinical samples for species detection and analysis of antibiotic resistance.

## Results and discussion

### Real-time analysis framework

Our real-time analysis framework consists of several of *streaming* programs communicating to each other via the network sockets or inter-process communication pipes provided by Unix-like operating systems. These programs typically take a sequence of items as input and process them every time a given small number of items arrive. They either retain only the relevant statistics of the data, or upon processing any data items, immediately forward only the necessary information to the downstream programs for further processing. This data processing methodology requires only a small memory footprint and hence is relevant for processing large amounts of data, especially real-time data from MinION sequencing.

We developed a number of auxiliary programs to facilitate setting up a real-time pipeline to analyse MinION sequencing data. These include scripts for setting up communication channels in a pipeline, thereby allowing the pipeline to be deployed on a high-performance computing cluster to scale with massive amounts of data. Programs for simple analyses of MinION sequencing data such as initial analysis (npReader [10]) and read-filtering on the basis of read length and read quality are also provided.

**Fig. 1:**
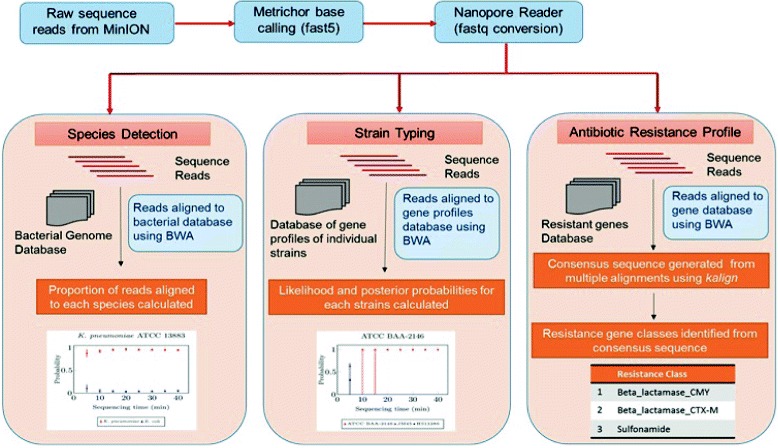
Schematic of the real-time analysis pipeline. Once the MinION starts sequencing, DNA fragments are sequenced (on the MinION) and base-called (by Metrichor cloud) instantaneously, and are simultaneously streamed through the pipeline where they are aligned by BWA-MEM [11]. *Arrows* show the data flow

We also developed streaming algorithms for a handful of identification problems, namely species typing, strain typing and identification of antibiotic resistance profiles (see Methods). We integrated the implementations of these algorithms into the analysis pipeline (see Fig. 1). In this pipeline, npReader [10] continuously scans the folder containing sequencing data in parallel with MinION sequencing. It picks up sequenced reads as soon as they are generated (from Metrichor), and simultaneously streams them through the pipeline for identification analyses. The pipeline also makes use of off-the-shelf bioinformatics tools such as BWA-MEM [11], as described later. In each step of this pipeline, data are piped from one process to the next without being written to disk, with the exception of base-calling via Metrichor in which each read is written to disk once it has been base-called, and is then picked up almost immediately by npReader.

We evaluated our real-time analysis pipeline and the accuracy of our algorithms using five MinION sequencing data sets. Four of these data sets were collected before the pipeline was developed, and hence we emulated the timing of the sequencing for the evaluation from these data sets. Specifically, we extracted the time that each read was sequenced, and streamed the sequence reads in the exact order and timing into the pipeline. With the emulation, we were able to stream the sequencing data at a hypothetical throughput 120 times higher than that we obtained with the MinION. This allowed us to test the scalability of the pipeline against the projected future throughput such as from the PromethION platform. The fifth data set was passed through our pipeline as it was base-called from Metrichor, and thus represents a true demonstration of the real-time capability of the pipeline. Finally, we validated the analysis results by sequencing these samples with the Illumina MiSeq platform, which has well-established bioinformatics analysis methods.

### Data generation

We prepared samples from cultured isolates of two *Klebsiella pneumoniae* strains ATCC BAA-2146, ATCC 13883; one *Klebsiella quasipneumoniae* strain ATCC 700603 and a library mixture sample. This mixture sample contained two different sequencing libraries prepared from the *Escherichia coli* strain ATCC 25922 and the *Staphylococcus aureus* strain ATCC 25923, pooled at different levels prior to sequencing (Table 1). We sequenced sample ATCC BAA-2146 and ATCC 700603 with the MinION using chemistry R7 and the others using the improved chemistry R7.3 (see Methods).

**Table 1:** Details of the four samples

Sample	Species	Strain	Information	Proportion
Single sample 1	*K. pneumoniae*	ATCC BAA-2146	NDM-1 positive resistant	100 %
Single sample 2	*K. quasipneumoniae*	ATCC 700603	K6, ESBL	100 %
Single sample 3	*K. pneumoniae*	ATCC 13883	Type strain	100 %
Mixture sample	*E. coli*	ATCC 25922	Seattle 1946	75 %
(Library mix)	*S. aureus*	ATCC 25923	Seattle 1945, Methicillin sensitive	25 %

To validate the analysis results from MinION sequencing, we sequenced all aforementioned isolates with the established Illumina platform MiSeq to a coverage exceeding 100-fold. Isolates in the mixture sample were sequenced separately. We assembled the MiSeq sequencing reads to obtain high quality assemblies of the five strains. With the assemblies, we were able to identify the sequence types and the antibiotic resistance profiles of these strains (see Methods). These results were used as the benchmarks to validate the analysis of MinION sequencing data.

### Sequencing yields and quality of MinION sequencing

**Fig. 2:**
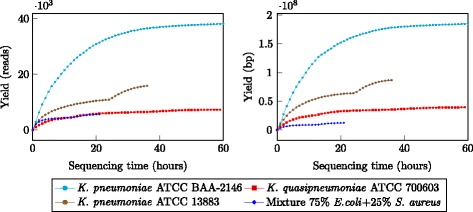
Sequencing yields over time for the four samples. Yields are shown in terms of read count (*left*) and base count (*right*)

Sequence reads from the MinION were classified into three types: *template*, *complement* and higher quality *2D* reads (*i.e.*, reads resulted from computationally merging a template and a complement read). The average Phred quality of template and complement reads across four runs was in the region of 5, while 2D reads were in higher quality, with average Phred quality about 9 (see Table 2 and Additional file 1: Figure S1). The median read lengths of three *K. pneumoniae* samples were approximately 5 Kb, while the mixture sample was only less than 1 Kb. We observed variation in terms of sequence yields across the four runs. While we obtained about 36 000 reads (185 Mb) for sample *K. pneumoniae* ATCC BAA-2146 after 60 h of sequencing, the run for sample *K. quasipneumoniae* ATCC 700603 yielded only 7092 reads (39 Mb) with the same running time (Fig. 2). We sequenced sample *K. pneumoniae* ATCC 13883 and the mixture sample for 36 and 20 h respectively, both with the chemistry 7.3, but the yields were markedly different. The read length and accuracy of our runs were consistent with other user reports [12–15].

**Table 2:** Details of the four MinION sequencing runs

Sample	Chemistry	Basecall	Time	Read	Base	Median	Quality	Quality 2D
		version	(hrs)	count	count (Mb)	length	mean (std)	mean (std)
Single sample 1	R7	1.4	60	38165	185	4580	4.70 (0.91)	8.96 (0.63)
Single sample 2	R7	1.4	60	7293	39	4936	4.95 (1.2)	9.34 (0.87)
Single sample 3	R7.3	1.9	36	15911	86	5242	4.58 (1.7)	9.46 (1.48)
Mixture sample	R7.3	1.10	21	5631	12	825	5.44 (2.1)	10.72(2.41)

Read quality in Phred score

### Species detection

For real-time bacterial species detection, we built a database from 2785 complete genomes of 1489 bacterial species available in GenBank (accessed Nov 2014), augmented with two *K. quasipneumoniae* genomes (which was not the strain we sequenced) as none were present in the database. The database contained several *K. pneumoniae*, *E. coli* and *S. aureus* strains (10, 63 and 49 respectively), but none of the five strains in our samples were present. The pipeline aligns sequence reads as they are generated from the sequencer to this database. The species typing algorithm periodically computes the simultaneous proportions of the species present in the sample and reports the 95 % confidence intervals of these proportions (see Methods).

**Fig. 3:**
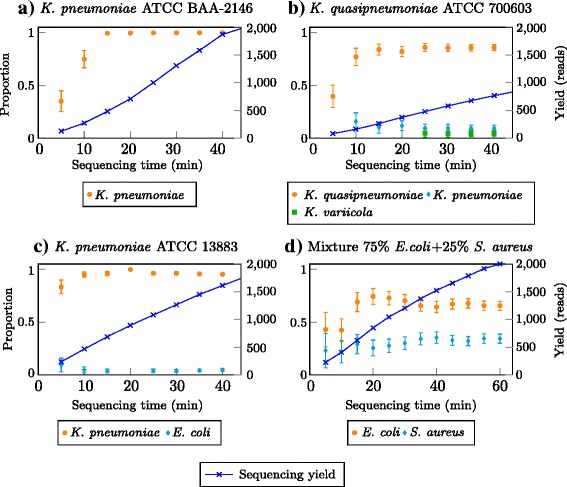
Real-time identification of bacterial species from MinION sequencing data for four different bacterial samples: a) *K. pneumoniae* ATCC BAA-2146, b) *K. quasipneumoniae* ATCC 700603, c) *K. pneumoniae* ATCC 13883 and d) Mixture of 75 % *E. coli* ATCC 25922 and 25 % *S. aureus* ATCC 25923. The *bars* represent confidence intervals at the 95 % level

In both *K. pneumoniae* samples as well as the *K. quasipneumoniae* sample, we successfully detected the major species present in the isolate. This was achieved with as little as 120 sequence reads requiring only 5 min of sequencing time (Fig. 3a, b and c). For *K. pneumoniae* strains ATCC BAA-2146 and ATCC 13883, it required less than 500 reads (10 and 15 min of sequencing, respectively) to reach a 95 % confidence interval of less than 0.05. For strain ATCC 700603 it required only 300 reads to correctly identify *K. quasipneumoniae* as the species.

The pipeline accurately identified the two species in the mixture sample as *E. coli* and *S. aureus* after obtaining around 100 reads (5 min of sequencing). The reported proportions became stable after around 1200 reads (35 min of sequencing). *E. coli* was the predominant species type in the mixture sample and it was evident with high proportion of sequencing reads supporting the *E. coli* species.

### Multi-locus sequence typing

Most bacteria are conventionally strain-typed using a multi-locus sequence typing (MLST) system that requires accurate genotyping to distinguish the alleles of seven house-keeping genes [16]. Our analysis of MinION raw read quality (Additional file 1: Figure S1), together with other user reports [12–15], indicated high error rates in MinION sequencing in comparison to Illumina Miseq sequencing. This suggested that MLST analysis would be challenging with MinION sequence data, especially in real-time fashion.

We developed a method to carry out MLST using MinION sequence data. Our method selected reads spanning each of the house-keeping genes. It then used multiple reads aligned to the same gene to correct error in the raw sequence reads and subsequently combined information across multiple alleles in a likelihood-based framework (see Methods). Table 3 presents the top five highest score sequence types (in log-likelihood) for *K. pneumoniae* and *K. quasipneumoniae* strains using MinION sequencing. In all three strains, the correct sequence types were the highest score out of 1678 sequence types available in the MLST database. We noticed that the typing system also outputted several other sequence types with the same likelihood (*e.g.*, ST-751 and ST-864 for strain ATCC BAA-2146 and ST-851 for strain ATCC 700603). We examined the profiles of these sequence types, and found them to be highly similar. For example, sequence types ST-751 and ST-864 (reported for strain ATCC BAA-2146) differed to the correct sequence type ST-11 by only one single nucleotide polymorphism (SNP) from the total of 3012 bases in seven genes. Similarly, sequence type ST-851 (co-highest score reported for strain ATCC 700603) differed to the correct sequence type ST-489 by two alleles (genes *phoE* and *tonB*). Because the run had a poor yield, only one read was aligned to these two genes by the end of the run, which may have also contributed to the inability to differentiate these two sequence types. While the results were encouraging, this also suggested that traditional MLST with nanopore sequencing requires high coverage to report the sequence type with absolute certainty. A more accurate strain-typing methodology would need to consider all of the sequenced reads, rather than just those 7 house-keeping genes. Therefore we further devised a method for strain-typing which was based on presence or absence of genes.

**Table 3:** MLST results for three *K. pneumoniae* strains

	ATCC BAA-2146		ATCC 700603		ATCC 13883	
	ST-11		ST-489		ST-3	
Rank	Type	Score	Type	Score	Type	Score
1	**ST-11**	**1985.47**	**ST-489**	**418.45**	**ST-3**	**1451.65**
2	**ST-751**	**1985.47**	**ST-851**	**418.45**	ST-136	1450.21
3	**ST-864**	**1985.47**	ST-257	413.57	ST-38	1444.81
4	ST-1080	1984.46	ST-356	413.57	ST-1106	1444.19
5	ST-1680	1982.62	ST-414	413.57	ST-931	1441.44

The top five probable sequence types are shown for each sample. The highest score sequence types are highlighted in bold

### Strain typing by presence or absence of genes

We developed a novel strain typing method to identify a known bacterial strain from the MinION sequence reads based on patterns of gene presence and absence. This approach is intended to rapidly identify the presence of a sequence type that has already been characterised, for example in an outbreak scenario, with subsequent confirmation using MLST once more data has been collected. We downloaded the genome assemblies of all strains for *K. pneumoniae*, *E. coli* and *S. aureus* species from the RefSeq repository and identified their sequence types using the relevant MLST schemes. This resulted in sets of 125 sequence types for *K. pneumoniae*, 353 for *E. coli* and 107 for *S. aureus*. For each sequence type, we picked the highest quality assembly (in terms of N50 statistics) and extracted gene sequences from its RefSeq gene annotation. We then grouped genes from a species based on 90 % sequence identity, and therein obtained the gene profile for each sequence type.

**Fig. 4:**
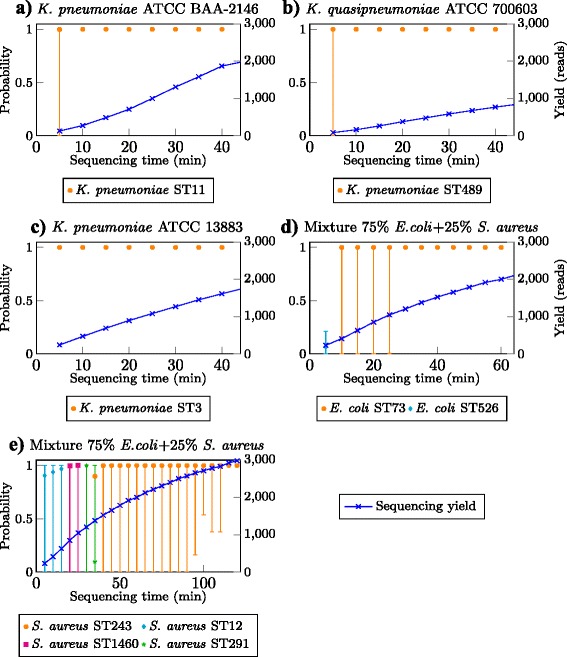
Real-time strain identification from MinION sequencing data on three different *K. pneumoniae* strains (**a**, **b** and **c**) and a *E. coli* strain (**d**) and a *S. aureus* strain (**e**) from the mixture sample. The *bars* represent confidence intervals at the 95 % level

Our pipeline identified genes present in the sample from sequence reads as they were generated by the MinION device. It then used this information to infer the posterior probability of each of the sequence types, as well as the 95 % confidence intervals in this estimate (see Methods). For our *K. pneumoniae* and *K. quasipneumoniae* samples, we successfully identified the corresponding sequence types from the sequence data with 95 % confidence within 10 min of sequencing time and with as few as 200 sequence reads (Fig. 4a, b and c). We streamed sequence reads from the mixture sample through the strain typing systems for *E. coli* and *S. aureus*, and in both cases, the correct sequence types of two species in the sample were also recovered. The correct sequence type for *E. coli* strain in the 75 %/ 25 % *E. coli*, *S. aureus* mixture was recovered after 25 min of sequencing with about 1000 total reads (or approximately 750 *E. coli* derived reads) (Fig. 4d). The pipeline was able to correctly predict the *S. aureus* strain (which is known to have much less gene content variation) in this mixture sample after 2 h of sequencing with about 2800 total reads (or approximately 700 *S. aureus* derived reads).

### Antibiotic resistance detection

The antibiotic resistance gene profiles of the samples were also characterised with MinION sequencing data. We obtained antibiotic drug resistance genes from the *ResFinder* database [17] (accessed July 2015). This set contained 2132 gene sequences, including variants of the same genes. We grouped these gene sequences based on 90 % sequence identity into 609 groups. In this grouping, we found that sequences in a group were variants of the same gene.

Our antibiotic resistance profile identification pipeline aligned sequence reads to this antibiotic gene database. The algorithms retained reads that aligned to these genes, and periodically performed multiple alignment of reads that were aligned to the same gene. It then generated a consensus sequence from these reads, and used a probabilistic Finite State Machine [18] to re-align the consensus sequence to the gene sequence (see Methods). The pipeline reported the presence of a resistance gene as soon as the alignment score reached a threshold.

Table 4 shows the time-line of antibiotic gene detection from MinION sequencing of three *K. pneumoniae* strains. For the NDM-1-producing strain ATCC BAA-2146, we identified the presence of 26 antibiotic resistance genes in the MiSeq assembly of the strain. Our real-time pipeline identified all these 26 genes and an additional gene *blaSHV* from 10 h of MinION sequencing. No further genes were detected thereafter. As gene *blaSHV* was reported with high confidence from the real-time analysis, we further investigated the alignment of the MiSeq assembly with this gene, and found that the gene was aligned to two contigs in the assembly suggesting the MiSeq assembly was fragmented in the middle of the gene. We sourced a high quality assembly of the strain’s genome using PacBio sequencing [19] and found that the assembly contained the gene. In other words, our pipeline detected precisely the antibiotic gene profile for this strain from 10 h of MinION sequencing. We observed that the majority of these genes were identified in the early stage of sequencing, *i.e.*, three quarters were reported within 1.5 h of sequencing, at fewer than 4000 reads (making up only a 3-fold coverage of the genome). We observed similar performance for *K. pneumoniae* strain ATCC 13883 where 5 out of 6 genes were detected after 2 h of sequencing. The last gene (*oqxB*) was detected after 9.5 h of sequencing, again recovering the full resistance profile without any false positive. For the multi-drug resistant *K. quasipneumoniae* strain ATCC 700603, the pipeline only detected 8 out of 11 genes. The reduced sensitivity for this sample was most likely due to the low sequence yield (33 Mb of data in total, or only 7-fold coverage of the genome).

**Table 4:** Time-line of resistance gene detection from the *K. pneumoniae* samples

Time	genes	Class	TP/FP	Sensitivity	Specificity	Data
(mins)				(%)	(%)	(no. of reads)
*K. pneumoniae* ATCC BAA-2146						
30						1228
	mphA	macrolide	TP			
	blaSHV	beta-lactamase	FP ^∗^			
	strA	aminoglycoside	TP			
	blaTEM	beta-lactamase	TP			
	strB	aminoglycoside	TP			
	blaCTX	beta-lactamase	TP	26.67	87.50	
60						2613
	blaLEN	beta-lactamase	TP			
	sul2	sulphonamide	TP			
	blaOXA	beta-lactamase	TP			
	aac3	aminoglycoside	TP			
	aac6	aminoglycoside	TP			
	blaCMY	beta-lactamase	TP			
	blaCFE	beta-lactamase	TP			
	blaLAT	beta-lactamase	TP			
	blaBIL	beta-lactamase	TP	53.33	94.12	
90						3844
	QnrB	quinolone	TP			
	aadA	aminoglycoside	TP			
	oqxA	quinolone	TP			
	tetA	tetracycline	TP			
	oqxB	quinolone	TP	76.67	95.83	
120						5258
	dfrA	trimethoprim	TP	80.00	96.00	
240						10 788
	blaOKP	beta-lactamase	TP	83.33	96.15	
270						11 931
	rmtC	aminoglycoside	TP	86.67	96.43	
300						13 022
	sul1	sulphonamide	TP			
	sul3	sulphonamide	TP	93.33	96.55	
540						20 200
	fosA	fosfomycin	TP	96.67	96.67	
600						21 546
	blaNDM	beta-lactamase	TP	100.00	96.77	
*K. quasipneumoniae* ATCC 700603						
30						582
	oqxA	quinolone	TP			
	blaSHV	beta-lactamase	TP			
	oqxB	quinolone	TP	27.27	100.00	
60						1090
	aadB	aminoglycoside	TP	36.36	100.00	
390						3704
	sul1	sulphonamide	TP			
	sul3	sulphonamide	TP	54.55	100.00	
420						3810
	blaOXA	beta-lactamase	TP	63.64	100.00	
540						4156
	blaOKP	beta-lactamase	TP	72.73	100.00	
*K. pneumoniae* ATCC 13883						
30						1264
	fosA	fosfomycin	TP	16.67	100.00	
60						2186
	blaSHV	beta-lactamase	TP			
	blaOKP	beta-lactamase	TP	50.00	100.00	
90						2952
	blaLEN	beta-lactamase	TP	66.67	100.00	
120						3584
	oqxA	quinolone	TP	83.33	100.00	
570						8112
	oqxB	quinolone	TP	100.00	100.00	

TP/FP: true positives/false positives according to the resistance gene profiles obtained from MiSeq sequencing. ^∗^Gene blaSHV was detected from MinION sequencing of *K. pneumoniae* ATCC BAA-2146 but not from MiSeq sequencing due to the inability to resolve a repeat in the gene

### Comparison with other methods

To date, only a few pipelines exist to identify species/subspecies from nanopore sequencing data, namely Metrichor [8, 20] and MetaPORE [9]. These methods commonly place the sample of question to a phylogeny taxonomy based on the number of reads that either are aligned to, or have a similar k-mer profile to, the taxon’s reference genome. Our species typing method is somewhat similar to this approach, although it additionally estimates confidence intervals in the species assignment. While we found that this approach can successfully identify species within 500 reads, the signal-to-noise from nanopore sequencing is too low to use a similar approach to correctly discriminate at the strain level, unless a large amount of data is available. Our strain typing uses a novel approach based on the presence and absence of genes and hence is able to make inference from a smaller number of reads.

Among the mentioned methods, only Metrichor [20] and MetaPORE [9] support genuine real-time analysis. As MetaPORE only focuses on viral species identification, we could only directly compare the performance of our method to Metrichor. We uploaded the first 1000 reads from our single samples and the first 3000 reads from our mixture sample to the Metrichor What’s In My Pot Bacteria k24 for SQK-MAP005 v1.27 (WIMP) workflow. Along with the species/subspecies and strains reported, WIMP provides a *classification score* filter where users can set the permissiveness of reporting. Table 5 presents the bacterial taxa reported by the WIMP workflow for our data with the default classification score. For sample *K. pneumoniae* ATCC BAA-2146, WIMP only returned the taxon *K. pneumoniae* at the species level. On the other hand, for the second and third samples (*K. quasipneumoniae* ATCC 700603 and *K. pneumoniae* ATCC 13883), WIMP reported several *K. pneumoniae* strains, but not the correct sequence types of these samples (ST489 and ST3). For the mixture sample, two *E. coli* and three *S. aureus* strains were reported, but these were also the incorrect sequence types (*E. coli* ST73 and *S. aureus* ST243). While it was unclear whether the sequence types of these samples were included in WIMP’s database, ST11 clearly was as it was reported in sample *K. pneumoniae* ATCC 700603. However, WIMP was unable to identify sample *K. pneumoniae* ATCC BAA-2146 to the strain level with 1000 reads, while our pipeline could do so in less than 400 reads (Fig. 4).

**Table 5:** Report of Metrichor What’s In My Pot Bacteria k24 for SQK-MAP005 v1.27 (WIMP) from the first 1000 reads of three single samples and the first 3000 reads of the mixture sample

Sample	Reported by Metrichor	Sequence type	Level	Accuracy
				species/strain
*K. pneumoniae* (ATCC BAA-2146, ST11)	*K. pneumoniae*	-	Species	$\checkmark /$
*K. quasipneumoniae* (ATCC 700603, ST489) ^∗^	*K. pneumoniae* subsp. pneumoniae	-	Sub-species	$\checkmark /$
	*K. pneumoniae* 342	ST146	Strain	*✓*/×
	*K. pneumoniae* JM45	ST11	Strain	*✓*/×
	*K. pneumoniae* CG43	ST86	Strain	*✓*/×
	*K. oxytoca*	-	Species	×/
	*K. variicola* At-22	-	Strain	*✓*/×
*K. pneumoniae* (ATCC 13883, ST3)	*K. pneumoniae* subsp. pneumoniae 1084	ST1084	Strain	*✓*/×
	*K. pneumoniae* CG43	ST86	Strain	*✓*/×
	*K. pneumoniae* subsp. rhinoscleromatis SB3432	ST67	Strain	*✓*/×
	*E. coli* O103:H2 str. 12009	ST17	Strain	×/ ×
Mixture sample	*E. coli* UMN026	ST597	Strain	*✓*/×
75 % *E. coli* (ATCC 25922, ST73)	*E. coli* ETEC H10407	ST48	Strain	*✓*/×
	*S. aureus* subsp. aureus HO 5096 0412	ST22	Strain	*✓*/×
25 % *S. aureus* (ATCC 25923, ST243)	*S. aureus* subsp. aureus MRSA252	ST36	Strain	*✓*/×
	*S. aureus* subsp. aureus T0131	ST239	Strain	*✓*/×
	*Yersinia pestis*	-	Species	×/

The last column indicates if the detection is correct ($\checkmark $) or incorrect (×) at species/strain levels. The Metrichor was able to identify the species (with some false positives) but not the strains in our samples

^*^ *K. quasipneumoniae* ATCC 700603 strain was recently re-classified from *K. pneumoniae* as *K. quasipneumoniae* [49] but has not been updated in most major databases

Our species typing module has some similarities to the approach used by MetaPhlAn [21], which was designed for metagenomics inference using millions of short-reads. Like MetaPhlAn, we used the proportion of reads that map to different taxonomic groupings to estimate the proportion of different species in a sample. MetaPhlAn optimises computational speed by aligning to a precomputed database of sequences that are pervasive within a single taxonomic grouping but not seen outside that grouping. This allows it to blast against a database that is 20 times smaller than a full bacterial genomic database. Our species typing approach, on the other hand, is designed to make a similar inference using only hundreds of reads, and moreover, also continuously updates confidence intervals so the user knows when they can stop sequencing and make a diagnosis.

Antibiotic resistance gene detection from MinION sequencing was also explored in Judge et al. [22]. Their approach was broadly similar to ours in that it initially aligned sequence reads to a resistance gene database, and then constructed a consensus sequence from the multiple alignment of matched reads. Both pipelines reported close to perfect resistance gene identification when compared with Illumina MiSeq sequencing. However, our pipeline uses a novel alignment parameter estimation using probabilistic Finite State Machines (see Methods). It is hence able to confidently report the presence of a resistance gene as soon as sufficient supporting data are available. This is the essence of real-time analysis presented here.

### Computational time

In our analyses, sequence reads were streamed through the pipeline in the exact order and timing that they were generated. Analysis results were generated periodically (every minute for species typing and strain typing and every five minutes for resistance gene identification). We examined the scalability of the pipeline to higher throughput by running the pipeline on a single computer equipped with 16 CPUs and streaming all sequence reads from the highest yield run (185 Mb from sample *K. pneumoniae* ATCC BAA-2146) through the pipeline at 120 times higher speed than they were generated (*e.g.*, data sequenced in 2 min were streamed within 1 s). Analysis results were generated every 5 s for typing and every one minute for gene resistance analysis. With this hypothetical throughput, our pipeline correctly identified the species and strain of the sample in less than 20 s; thereupon we could terminate the typing analyses. The pipeline then reported all the resistance genes in five minutes, which corresponded to the data generated in the first 10 h of actual sequencing. This demonstrates the scalability of our pipeline to higher throughput sequencing platforms in the future.

### Real-time analysis of a clinical isolate

With the pipeline in place, we analysed a clinical *K. pneumoniae* isolate collected in Greece that was found to be resistant to an extensive range of antibiotics. We sequenced the sample on the MinION with Chemistry R7.3 and ran the Metrichor service, which performed basecalling and sample identification during the first three hours of the run. We also ran our pipeline in real-time on the base-called data returned from the Metrichor service.

**Fig. 5:**
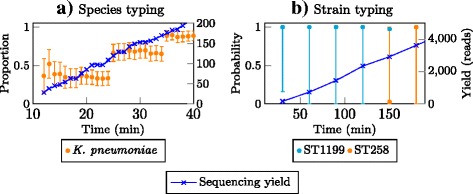
Real-time species typing (**a**) and strain typing (**b**) of a clinical isolate directly from the MinION using our pipeline and the Metrichor service. The time includes basecalling timing

We observed a delay from the base-calling of the data; the first read was sequenced on the MinION within one minute from starting the run, but the base-called data were received after 6 min. The delay tended to increase as more data were generated. We found the base-called data returned during the three-hour run of the Metrichor service were actually sequenced within 45 min on the MinION. This highlights the need for a local base-calling step to improve real-time analysis. Figure 5a and 5b show the timing (from the start of the MinION run) of sample identification using our pipeline. The pipeline reported *K. pneumoniae* as the only species in the sample within 10 min, and reached a confidence interval of less than 0.1 in 40 min when approximately 200 reads were analysed. We noticed that these 200 reads were actually sequenced in 7 min by the MinION. For strain identification, our pipeline initially reported ST1199 but after 2.5 h, reported ST258 as the sequence type for this isolate. It is worth noting that the two strains are highly similar; their MLST profiles differ by only one SNP in the seven house-keeping genes. By sequencing the isolate on the Illumina MiSeq as described above, we confirmed that the sequence type for the strain is ST258. On the other hand, the sample identification from Metrichor initially reported *K. pneumoniae* 1084 (ST23), but finally reported two strains namely *K. pneumoniae* JM45 (ST11) and *K. pneumoniae* HS11286 (ST11) after 3 h (Additional file 2: Figure S2). During the three-hour run with less than 4000 reads (16 Mb of data), our pipeline reported two antibiotic resistance genes, namely *sul2* (sulphonamide) and *tetA* (tetracycline). Our analysis of the Illumina data for this strain confirmed the presence of these two genes. Clinical susceptibility testing also showed the resistance of this isolate to tetracycline and sulfamethoxazole-trimethoprim (MIC ≥ 16 *μ*g/mL and ≥ 320 *μ*g/mL, respectively analyzed by VITEK®;2 bioMérieux, Inc). Finally, we re-analysed the data from this run using the emulation described previously, and obtained the same results as from the real-time analysis.

### Discussion

In recent years high-throughput sequencing has become an integrative tool for infectious disease research [23, 24], predominantly using massively parallel short-read sequencing technologies. These technologies achieve a very high base calling accuracy, making them ideally suited to applications requiring accurate calling of SNPs. However, these technologies attain their high yield by sequencing a single base per cycle for millions of sequence fragments in parallel, where each cycle takes at least 5 min.

The Oxford Nanopore MinION device, on the other hand, generated as many as 500 reads in the first 10 min of sequencing in our hands (which is 3 times lower than the theoretical maximum). The error rate of these reads was substantially higher than the corresponding Illumina short-read data. Many existing bioinformatics algorithms rely on accurate base and SNP calling, which makes their application to MinION data challenging. As an example, most existing strain typing approaches often use a MLST system, either on a pre-defined set of house keeping genes [25], or on core genes set [26]. These approaches are highly standardised, reproducible and portable, and hence are routinely used in laboratories around the world. Rapid genomic diagnosis tools using MLST from high-throughput sequencing such as SRST2 [27] have also been developed. While we showed in this article that MLST can be adapted to identify bacterial strains from nanopore sequencing, this requires high coverage sequencing of the gene set to overcome the high error rates.

The main contribution of this article is to demonstrate that despite the higher error rate, it is possible to return clinical actionable information, including species and strain identification from as few as 500 reads. We achieved this by developing novel approaches that are less sensitive to base-calling errors and which use whatever subset of genome-wide information is observed up to a point in time, rather than a panel of pre-defined markers or genes. For example, the strain typing presence/absence approach relies only on being able to identify homology to genes and also allows for a level of incorrect gene annotation.

Our strain typing module has the advantage of being able to rapidly type a known strain with a small number of low quality (*i.e.*, mostly 1D) reads. Competing approaches using k-mers appear to require substantially more high quality data. The drawback of our approach is that if a large number of genes are lost or gained in a single event, such as the gain or loss of a plasmid, the most likely strain may be incorrect. Thus it would be ideally suited for rapidly typing a known strain in an outbreak scenario.

Our antibiotic resistance module is able to identify the drug resistance potential of an isolate within a few hours of sequencing with very high specificity. In particular, with the most recent chemistry utilised in this paper (R7.3), we were able to identify the complete resistance potential of a *K. pneumoniae* isolate without any false positives in 9.5 h, and with approximately 8000 reads, (80 % of the resistance genes were identified with 3000 reads in 2 h). In order to achieve high specificity we designed a probabilistic Finite State Machine for error correction.

One of the major advantages of a whole-genome sequencing approach to drug resistance profiling is that it is not necessary to restrict the analysis to a limited panel of drug-resistance tests, but it is possible to discover the complete drug resistance profile in a sample. With a complete picture of the drug-resistance profile within a few hours, a clinician may be able to design an antibiotic treatment regimen that is both more likely to succeed and less likely to induce further antibiotic resistance. However, even achieving completely accurate identification of resistance genes is only a first step in accurately predicting the resistance profile, as mutations may effect the rate at which these genes are transcribed and also their antibiotic resistance activity. Prediction of antibiotic resistance from genotype is an area that warrants substantial further research.

In summary, we have developed an open-source, flexible pipeline for real-time analysis of MinION sequencing data. The pipeline includes various streaming algorithms to identify pathogens and their antibiotic resistance, but others can be seamlessly integrated into [28]. The only step in our pipeline at which data are written to, and then re-read from disk is the base-calling step using Metrichor. npReader immediately identifies new reads as they are generated by Metrichor; however, some delay can occur while waiting for base-called data to be returned from Metrichor. Oxford Nanopore Technologies have recently opened up the Application Programming Interface to extract raw data directly from the MinION. This, together with the recent development of open source base-calling algorithms [29, 30] to run on the local machine, will allow future development of a completely streaming pipeline, in the sense of never saving data to disk. Our pipeline can be deployed on a single 16 core computer, capable of analysing MinION data streaming at up to 120 × the current rate of sequencing; or on a high performance computing cluster to scale with the potential even higher throughput of forthcoming nanopore sequencing platforms.

## Methods

### DNA extraction

Bacterial strains *K. pneumoniae* ATCC BAA-2146, ATCC 13883, *K. quasipneumoniae* ATCC 700603, *E. coli* ATCC 25922 and *S. aureus* ATCC 25923 were obtained from the American Type Culture Collection (ATCC, USA). *K. pneumoniae* clinical isolate was acquired from Hygeia General Hospital, Athens, Greece from a patient stool sample in 2014 (Lab ID 100575214, isolate 1). Clinical susceptibility profiling by VITEK®;2 (bioMérieux Inc.) identified the isolate as carbapenemase-producing (KPC), giving rise to extended spectrum *β*-lactam resistance. It was also deemed resistant to aminoglycoside, phenicol, quinolone, sulphonamide, tetracycline and trimethoprim antibiotics, rendering it an extensively drug-resistant bacterial isolate. Bacterial cultures were grown overnight from a single colony at 37 °C with shaking (180 rpm). Whole cell DNA was extracted from the cultures using the DNeasy Blood and Tissue Kit (QIAGEN Ⓒ, Cat #69504) according to the bacterial DNA extraction protocol with enzymatic lysis pre-treatment.

### MinION sequencing

Library preparation was performed using the Genomic DNA Sequencing kit (Oxford Nanopore) according to the manufacturer’s instruction. For the R7 MinION Flow Cells SQK-MAP-002 sequencing kit was used and for R7.3 MinION Flow Cells SQK-MAP-003 or SQK-MAP-006 Genomic Sequencing kits were used according to the manufacturer’s instruction.

For the library mixture sample, the DNA concentration of each library was measured using Qubit Fluorimeter (Thermo Fisher Scientific). Based on the concentration, 75 % of *E. coli* (ATCC 25922) library and 25 % of *S. aureus* (ATCC 25923) library were mixed prior to sequencing.

A new MinION Flow Cell (R7 or R7.3) was used for sequencing each sample. The library mix was loaded onto the MinION Flow Cell and the Genomic DNA 48 h sequencing protocol was initiated on the MinKNOW software.

### MinION data analysis

The sequence read data were base-called with Metrichor Agent. We used npReader [10] to convert base-called sequence data in fast5 format to fastq format. The npReader program also extracted the time that each read was sequenced and used this information to sort the read sequences in order they were produced. For the real-time analyses, we wrote a program to emulate the sequencing process in that it streamlined each read in the exact order it was sequenced. The program also allowed us to scale up the sequencing emulation to a factor of choice. Our pipeline allows for filtering out 1D reads at multiple stages (including via npReader). All subsequent analyses in this paper used both 1D and 2D reads.

### MiSeq sequencing and data analysis

Library preparation was performed using the NexteraXT DNA Sample preparation kit (Illumina), as recommended by the manufacturer. Libraries were sequenced on the MiSeq instrument (Illumina) with 300 bp paired end sequencing, to a coverage of over 100-fold. Read data were trimmed with *trimmomatic* [31] (V0.32) and subsequently assembled using SPAdes [32] (V3.5), resulting in assemblies with N50 exceeding 200 Kb. Their sequence types were identified by submitting the assembled genomes to the MLST servers [33] for *K. pneumoniae*, *E. coli* (set #1) and *S. aureus*.

We identified the antibiotic resistance profiles of these strains from their MiSeq assemblies. We used blastn (V2.29)to align these assemblies to the database of resistance genes obtained from ResFinder [17]. Genes that were covered at least greater than 85 % by the alignments and with greater than 85 % sequence identity were considered to be present in the sample. These gene profiles were used as a benchmark to validate the MinION sequencing analysis.

### Species typing

We downloaded the bacterial genome database on GenBank (accessed 19 Nov 2014), which contained high quality complete genomes of 2785 bacterial strains from 1487 bacteria species. We expanded this database to include two *K. quasipneumoniae* genomes. Our species typing pipeline streamed read data from npReader directly to BWA-MEM [11] (V0.7.10-r858), which aligned the reads to the database. Output from BWA in SAM format was streamed directly into our species typing pipeline, which calculated the proportion of reads aligned to each of these species. Our species typing method considers the proportions {*p*_1_,*p*_2_,..,*p*_*k*_} of *k* species in the mixture as the parameters of a *k*-category multinomial distribution, and the read counts {*c*_1_,*c*_2_,..,*c*_*k*_} for the species as an observation from *c*_1_+*c*_2_+..+*c*_*k*_ independent trials drawn from the distribution. It then uses the MultinomialCI package in R [34] to calculate the 95 % confidence intervals of these proportions from the observation.

### Multi-locus sequence typing

MinION sequence reads from *K. pneumoniae* strains were aligned to the seven house-keeping genes specified by the MLST system using BWA-MEM [11]. We then collected reads that were aligned to a gene and performed a multiple alignment on them using kalign2 [35]. The consensus sequence created from the multiple alignment was then globally aligned to all alleles of the gene using a probabilistic Finite State Machine (see below) for global alignment. The score of a sequence type was determined by the sum of the scores of seven alleles making up the type.

### Strain typing

We built gene profile databases for *K. pneumoniae*, *E. coli* and *S. aureus* from the RefSeq annotation. Specifically, we obtained the publicly available assemblies of these species listed on the RefSeq (accessed 17 July 2015). We used the relevant MLST schemes obtained from [33] to identify sequence type of each assembly. For each sequence type, we selected the assembly with highest N50 statistic and use the RefSeq gene annotation of the assembly to determine the gene content of the sequence type.

In order to develop a simple probabilistic presence/absence strain typing model, we considered the genomes of each of the strains simply as a collection of genes. Denote by *St*_*j*=1..*J*_ all the strains in our database (for a fixed species). Denote by *g*_*j*,*k*_ the *k*^*th*^ gene in the database for strain *j*, where the genes are listed in no particular order. Denote by *N*_*j*_ the total number of genes in *St*_*j*_.

We aligned each sequence read *r*_*i*_ from the MinION device to the gene database using BWA-MEM [11]. We counted the number of genes of each strain that aligned to read *r*_*i*_, denoted by *N*_*j*_(*r*_*i*_).

We describe below how to calculate the likelihood, *P*(*r*_*i*_|*St*_*j*_), of each strain generating each read, from which we can calculate the posterior probability of each strain *St*_*j*_ conditional on observing the reads *r*_1_…*r*_*m*_:

1 $$ P({St}_{j}|r_{1}..r_{m}) = \frac{\prod_{i=1..m}P(r_{i}|{St}_{j})}{\sum_{j'}\prod_{i=1..m}P(r_{i}|{St}_{j})}  $$

The probability *P*(*r*_*i*_|*St*_*j*_) could be calculated using a simple model as:

2 $$ P_{\text{simple}}(r_{i} | {St}_{j}) = \frac{N_{j}(r_{i})}{N_{j}},  $$

However, this model suffers from the problem that if we observe any read that overlaps a gene not in the reference genome for *St*_*j*_, then the posterior probability of that strain will become zero. Thus, this model is very unstable. In order to make this estimate more stable, we used a mixture model that allows the read to have been generated by a background model:

3 $$ \begin{aligned} P(r_{i} | {St}_{j}) = (1-c)*\frac{N_{j}(r_{i})}{N_{j}} + (c)*P\left(r_{i} | \bigcup_{j'} {St}_{j'}\right). \end{aligned}  $$

The background model considers the probability that the read was generated from any of the strains:

4 $$  P\left(r_{i} | \bigcup_{j'} {St}_{j'}\right) = \frac{\sum_{j'} N_{j'}(r_{i})}{\sum_{j'} N_{j'}}.  $$

This makes the posterior probability estimates more stable. It also makes the model robust to incorrect annotation of the reads from the MinION sequencer and incorrect annotation of the reference genome. We have investigated use of *c*=0.2, *c*=0.1 and *c*=0.05 and found that it has little impact on the results, with slightly smaller confidence intervals (data not shown). We chose *c*=0.2 in order to conservatively estimate confidence intervals.

Finally, in order to calculate confidence intervals we employed a bootstrap resampling approach in which we resampled *m* reads from *r*_1_,…*r*_*m*_ with replacement. This is repeated 1000 times, and the posterior probabilities are recalculated every iteration. We calculated the 95 % confidence intervals from the empirical distribution of these posterior probabilities.

To gain some insight into how this model works in response to gene presence, consider a gene *g*, which is present in a fraction *f* of strains, including *St*_*j*_ but not including *St*_*k*_. For simplicity, assume that each strain has *N* genes. The difference in log-likelihood *St*_*j*_ and *St*_*k*_ conditional on *g* can be approximated by log(1/*c*)+ log(1/*f*), showing that a more specific gene has a stronger effect in our model than a common gene in distinguishing strains.

To gain insight into the effect of gene absence in contrast to gene presence, assume instead that the only difference between *St*_*j*_ and *St*_*k*_ is the deletion of a single gene (*g*) in *St*_*j*_, and denote by *N*=*N*_*j*_=*N*_*k*_−1. If we sequence *N* ln(2) genes from *St*_*j*_ without seeing gene *g*, the difference in log-likelihood becomes *N* ln(2)∗(log(*N*)− log(*N*−1))≈1bit, corresponding to the likelihood that *St*_*j*_ is twice as big as the likelihood of *St*_*k*_. For example, if a strain has 1000 genes, then we would need to observe 693 genes without observing *g* to be able to conclude that the observed data were twice as likely to be generated from the species with a single gene deletion. For comparison, we would need to only sequence 100 genes from *St*_*k*_ to get an expected log-likelihood difference of 1 bits versus *St*_*j*_, demonstrating the extra information in gene ’presence’ versus ’absence’ typing.

### Antibiotic resistance gene classes detection

We downloaded the resistance gene database from ResFinder [17] (accessed July 2015). We aligned each gene to the collection of bacterial genomes in RefSeq using blastn [36], and used the best alignment of the gene to extract 100 bp sequences flanking the antibiotic resistance genes. We found that the inclusion of these flanking sequences improved the sensitivity of mapping MinION reads to the gene database.

We then grouped these genes based on 90 % sequence identity into 609 groups. We manually checked and found that genes within a group were variants of the same gene. We selected the longest gene in each group to make up a reduced resistance gene database. To create a benchmark of resistance genes for a sample, we used blastn to compare the Illumina assembly of the sample against this reduced gene database, and reported genes with greater than 85 % coverage and identity.

Our analysis pipeline aligned MinION sequencing data to this reduced resistance gene database using BWA-MEM [11] in a streamlined fashion, and examined genes with reads mapping to the whole gene (not including flanking sequences). Because of high error rates with MinION sequence data, we noticed a high rate of false positive genes. To reduce false positives, we used kalign2 [35] to perform a multiple alignment of reads that were aligned to the same gene. The consensus sequence resulting from the multiple alignment was then compared with the gene sequence using a probabilistic Finite State Machine (see below). The pipeline then reported gene classes based on the genes detected.

### Sensitive alignment of noisy sequences with probabilistic Finite State Machines

Our methods for MLST strain typing and antibiotic resistance gene identification require the alignment of a consensus sequence to a gene or a gene allele. Such an alignment generally assumes a model and a set of parameters of the differences between the sequences. It is widely recognised that the accuracy of the alignment is sensitive to these parameters [37–39]. However, in the context of real-time analysis of MinION sequencing, it is not possible to select in advance a sensible set of parameters. On the one hand, the quality among sequence reads differs remarkably; as shown in Additional file 1: Figure S1 and Table 2 – the majority (95 %) of the reads across our four runs have a Phred score ranging between 3 and 7 for template and complement reads (corresponding to 50–80 % accuracy) and between 6 to 12 for 2D reads (75–95 % accuracy). On the other hand, a consensus sequence is computationally constructed from a set of reads. Its quality is hence contingent to not only the quality of the reads but also the number of reads in the set.

**Fig. 6:**
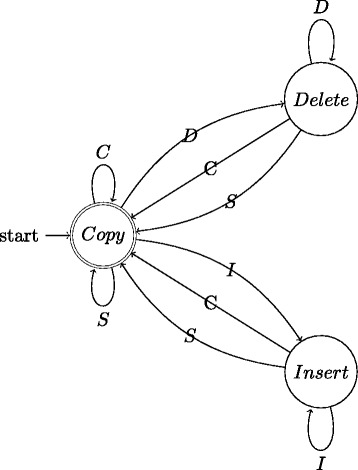
Schematic of a three-state probabilistic Finite State Machine

We use a probabilistic Finite State Machine (pFSM) [40] to model the differences, and hence the simultaneous error profile of the consensus sequence. Briefly, a pFSM is a probabilistic model of genomic alignment that takes into account different types of variations including SNPs, insertions and deletions. A pFSM is equivalent to a hidden Markov Model. The pFSM consists of a set of states and transitions between states. Each transition corresponds to an *action* and is associated with a cost for the action. An action could be one of *copy* (*C*), *substitute* (*S*), *delete* (*D*) and *insert* (*I*). Figure 6 depicts a three-state pFSM, which is equivalent to an affine gap penalty alignment model. In order to assess an alignment of two sequences A and B, under a hypothesis specified by the parameters, the pFSM computes the cost to generate one sequence (say A) given the other (B). For example, while in state *Copy*, the machine consumes the next base in B, generates the next base in A; it is said to take action *C* if the two bases are the same, or action *S* otherwise, and to follow either transition to state *Copy*. Alternatively, the machine can take either action *D* (consumes the next base in B without generating any base in A and moves to state *Delete*), or action *I* (generates the next base in A without consuming a base in B and moves to state *Insert*). These actions are repeated until the whole sequence B is generated.

We used an information-theoretic measure whereby the cost of a transition is that of encoding the generated base, or in other words, the negative logarithm of the probability of the associated action (*c*=−*log*_2_(*P*(*a*)). The foundation of this approach goes back to the 1960s when it was proposed as a basis for inductive inference [41, 42]. It has since been used in several bioinformatics applications such as for calculating the BLOSUM matrix [43] and modelling DNA sequences [44, 45]. More importantly, this information-theoretic framework allows one to estimate a sensible set of parameters for any *related* two sequences. This is done via an Expectation-Maximisation process. This starts with an initial set of probabilities at each state. In the E-step, the best alignment (lowest cost) is calculated by a dynamic programming algorithm. The frequencies of actions at each state are then used to re-estimate the probabilities in the M-step. A detailed discussion of this process is provided in Allison *et al* [40] and Cao et al. [46]. The process is guaranteed to converge to an optimal, and it does so in only a few iterations in our experience.

## Abbreviations

MLST, multi-locus sequence typing; pFSM, probabilistic Finite State Machine

## Additional files

Additional file 1
**Figure S1.** Histograms of read quality (in Phred score) and read lengths of four MinION sequencing runs. (PDF 112 kb)

Additional file 2
**Figure S2.** Screen shot of What’s In My Pot (WIMP) analysis of the clinical sample after three hours of sequencing. (PNG 58 kb)

## References

[1] Boyd SD (2013). Diagnostic applications of high-throughput DNA sequencing. Ann Rev Pathol.

[2] Koboldt DC, Steinberg KM, Larson DE, Wilson RK, Mardis ER (2013). The next-generation sequencing revolution and its impact on genomics. Cell.

[3] Gaber MM, Zaslavsky A, Krishnaswamy S (2005). Mining data streams. ACM SIGMOD Record.

[4] Muthukrishnan S (2005). Data Streams: Algorithms and Applications. Foundations Trends Theor Comput Sci.

[5] Kasianowicz JJ, Brandin E, Branton D, Deamer DW (1996). Characterization of individual polynucleotide molecules using a membrane channel. Proc Nat Acad Sci.

[6] Branton D, Deamer DW, Marziali A, Bayley H, Benner SA, Butler T, Di Ventra M, Garaj S, Hibbs A, Huang X, Jovanovich SB, Krstic PS, Lindsay S, Ling XS, Mastrangelo CH, Meller A, Oliver JS, Pershin YV, Ramsey JM, Riehn R, Soni GV, Tabard-Cossa V, Wanunu M, Wiggin M, Schloss JA (2008). The potential and challenges of nanopore sequencing. Nat Biotechnol.

[7] Stoddart D, Heron AJ, Mikhailova E, Maglia G, Bayley H (2009). Single-nucleotide discrimination in immobilized DNA oligonucleotides with a biological nanopore. Proc Nat Acad Sci USA.

[8] Quick J, Ashton P, Calus S, Chatt C, Gossain S, Hawker J, Nair S, Neal K, Nye K, Peters T, De Pinna E, Robinson E, Struthers K, Webber M, Catto A, Dallman TJ, Hawkey P, Loman NJ (2015). Rapid draft sequencing and real-time nanopore sequencing in a hospital outbreak of Salmonella. Genome Biol.

[9] Greninger AL, Naccache SN, Federman S, Yu G, Mbala P, Bres V, Stryke D, Bouquet J, Somasekar S, Linnen JM, Dodd R, Mulembakani P, Schneider BS, Muyembe-Tamfum JJ, Stramer SL, Chiu CY (2015). Rapid metagenomic identification of viral pathogens in clinical samples by real-time nanopore sequencing analysis. Genome Med.

[10] Cao MD, Ganesamoorthy D, Cooper MA, Coin LJM (2016). Realtime analysis and visualization of MinION sequencing data with npReader. Bioinformatics.

[11] Li H. Aligning sequence reads, clone sequences and assembly contigs with BWA-MEM. 2013. 1303.3997#.

[12] Quick J, Quinlan AR, Loman NJ (2014). A Reference Bacterial Genome Dataset Generated on the {MinION} Portable Single-molecule Nanopore Sequencer. GigaScience.

[13] Ashton PM, Nair S, Dallman T, Rubino S, Rabsch W, Mwaigwisya S, Wain J, O’Grady J (2015). MinION nanopore sequencing identifies the position and structure of a bacterial antibiotic resistance island. Nat Biotechnol.

[14] Kilianski A, Haas JL, Corriveau EJ, Liem AT, Willis KL, Kadavy DR, Rosenzweig CN, Minot SS. Bacterial and viral identification and differentiation by amplicon sequencing on the MinION nanopore sequencer. GigaScience. 2015;4(1). doi:10.1186/s13742-015-0051-z. 10.1186/s13742-015-0051-z PMC4374364 25815165

[15] Jain M, Fiddes IT, Miga KH, Olsen HE, Paten B, Akeson M (2015). Improved data analysis for the MinION nanopore sequencer. Nat Methods.

[16] Diancourt L, Passet V, Verhoef J, Grimont PAD, Brisse S (2005). Multilocus Sequence Typing of Klebsiella pneumoniae Nosocomial Isolates. J Clin Microbiol.

[17] Zankari E, Hasman H, Cosentino S, Vestergaard M, Rasmussen S, Lund O, Aarestrup FM, Larsen MV (2012). Identification of Acquired Antimicrobial Resistance Genes. J Antimicrobial Chemother.

[18] Allison L, Wallace CS, Yee CN. When is a string like a string? In: Artificial Intelligence and Mathematics.1990. Ft. Lauderdale FL.

[19] Poznik DG, Henn BM, Yee MC, Sliwerska E, Euskirchen GM, Lin AA, Snyder M, Quintana-Murci L, Kidd JM, Underhill PA, Bustamante CD (2013). Sequencing {Y} Chromosomes Resolves Discrepancy in Time to Common Ancestor of Males Versus Females. Science.

[20] Juul S, Izquierdo F, Hurst A, Dai X, Wright A, Kulesha E, Pettett R, Turner DJ. What’s in my pot? Real-time species identification on the MinION. bioRxiv. 2015. doi:10.1101/030742.

[21] Segata N, Waldron L, Ballarini A, Narasimhan V, Jousson O, Huttenhower C (2012). Metagenomic microbial community profiling using unique clade-specific marker genes. Nat Methods.

[22] Judge K, Harris SR, Reuter S, Parkhill J, Peacock SJ (2015). Early insights into the potential of the Oxford Nanopore MinION for the detection of antimicrobial resistance genes. J Antimicrobial Chemother.

[23] Dunne WM, Westblade LF, Ford B (2012). Next-generation and whole-genome sequencing in the diagnostic clinical microbiology laboratory. Eur J Clin Microbiol Infect Dis Off Publ Eur Soc Clin Microbiol.

[24] Fricke WF, Rasko DA (2014). Bacterial genome sequencing in the clinic: bioinformatic challenges and solutions. Nat Rev Genet.

[25] Maiden MC, Bygraves JA, Feil E, Morelli G, Russell JE, Urwin R, Zhang Q, Zhou J, Zurth K, Caugant DA, Feavers IM, Achtman M, Spratt BG (1998). Multilocus sequence typing: a portable approach to the identification of clones within populations of pathogenic microorganisms. Proc Nat Acad Sci USA.

[26] Cody AJ, McCarthy ND, Jansen van Rensburg M, Isinkaye T, Bentley SD, Parkhill J, Dingle KE, Bowler ICJW, Jolley KA, Maiden MCJ (2013). Real-Time Genomic Epidemiological Evaluation of Human Campylobacter Isolates by Use of Whole-Genome Multilocus Sequence Typing. J Clin Microbiol.

[27] Inouye M, Dashnow H, Raven LA, Schultz MB, Pope BJ, Tomita T, Zobel J, Holt KE (2014). SRST2: Rapid genomic surveillance for public health and hospital microbiology labs. Genome Med.

[28] Cao MD, Nguyen SH, Ganesamoorthy D, Elliott A, Cooper M, Coin LJM. Scaffolding and Completing Genome Assemblies in Real-time with Nanopore Sequencing. BioRxiv. 2016. 054783. doi:10.1101/054783. 10.1038/ncomms14515 PMC5321748 28218240

[29] David M, Dursi LJ, Yao D, Boutros PC, Simpson JT. Nanocall: An Open Source Basecaller for Oxford Nanopore Sequencing Data. BioRxiv. 2016. 046086. doi:10.1101/046086. 10.1093/bioinformatics/btw569 PMC5408768 27614348

[30] Boža V, Brejová B, Vinar T. DeepNano: Deep Recurrent Neural Networks for Base Calling in MinION Nanopore Reads. 2016. 1603.09195. 10.1371/journal.pone.0178751 PMC5459436 28582401

[31] Bolger AM, Lohse M, Usadel B (2014). Trimmomatic: a flexible trimmer for Illumina sequence data. Bioinformatics.

[32] Bankevich A, Nurk S, Antipov D, Gurevich AA, Dvorkin M, Kulikov AS, Lesin VM, Nikolenko SI, Pham S, Prjibelski AD, Pyshkin AV, Sirotkin AV, Vyahhi N, Tesler G, Alekseyev MA, Pevzner PA (2012). SPAdes: A New Genome Assembly Algorithm and Its Applications to Single-Cell Sequencing. J Comput Biol.

[33] Larsen MV, Cosentino S, Rasmussen S, Friis C, Hasman H, Marvig RL, Jelsbak L, Sicheritz-Pontén T, Ussery DW, Aarestrup FM, Lund O (2012). Multilocus Sequence Typing of Total-Genome-Sequenced Bacteria. J Clin Microbiol.

[34] Sison CP, Glaz J (1995). Simultaneous Confidence Intervals and Sample Size Determination for Multinomial Proportions. J Am Stat Assoc.

[35] Lassmann T, Frings O, Sonnhammer ELL (2009). Kalign2: High-performance Multiple Alignment of Protein and Nucleotide Sequences Allowing External Features. Nucleic Acids Res.

[36] Altschul SF, Gish W, Miller W, Myers EW, Lipman DJ (1990). Basic Local Alignment Search Tool. J Mol Biol.

[37] Gusfield D, Balasubramanian K, Naor D (1994). Parametric Optimization of Sequence Alignment. Algorithmica.

[38] Frith M, Hamada M, Horton P (2010). Parameters for Accurate Genome Alignment. BMC Bioinformatics.

[39] Cao MD, Dix TI, Allison L (2010). A genome alignment algorithm based on compression. BMC Bioinformatics.

[40] Allison L, Wallace CS, Yee CN (1992). Finite-state models in the alignment of macromolecules. J Mol Evol.

[41] Solomonoff R (1964). A Formal Theory of Inductive Inference. Inform Control.

[42] Wallace CS, Boulton DM (1968). An Information Measure for Classification. Comput J.

[43] Henikoff S, Henikoff JG (1992). Amino acid substitution matrices from protein blocks. Proc Nat Acad Sci.

[44] Cao MD, Dix TI, Allison L, Mears C (2007). A simple statistical algorithm for biological sequence compression. Data Compression Conference.

[45] Cao MD, Dix TI, Allison L, Arabnia HRR, Tran Q-N (2011). A biological compression model and its applications. Software Tools and Algorithms for Biological Systems. Advances in Experimental Medicine and Biology.

[46] Cao MD, Dix TI, Allison L, Theeramunkong T, Kijsirikul B, Cercone N, Ho T-B (2009). Computing substitution matrices for genomic comparative analysis. Advances in Knowledge Discovery and Data Mining. Lecture Notes in Computer Science.

[47] Cao MD. Java package for sequence analysis. 2015. https://github.com/mdcao/japsa.

[48] Cao MD, Ganesamoorthy D, Elliott A, Zhang H, Cooper M, Coin L. Support data for “Streaming algorithms for identification of pathogens and antibiotic resistance potential from real-time MinION sequencing”. GigaScience Database. 2016. doi:10.5524/100206. 10.1186/s13742-016-0137-2 PMC4960868 27457073

[49] Elliott AG, Ganesamoorthy D, Coin L, Cooper MA, Cao MD (2016). Complete genome sequence of klebsiella quasipneumoniae subsp. similipneumoniae Strain ATCC 700603. Genome Announcements.

