# Supplementary material for: Streaming algorithms for identification of pathogens and antibiotic resistance potential from real-time MinIONTM sequencing
Source: Gigascience. 2016 Jul 26;5(1):32. doi: 10.1186/s13742-016-0137-2 (PMC4960868; doi:10.1186/s13742-016-0137-2)

(a) *K. pneumoniae* ATCC BAA-2146

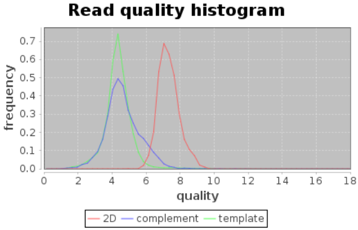

(b) *K. quasipneumoniae* ATCC 700603

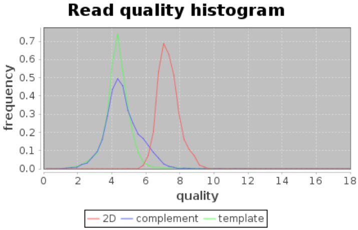

(c) *K. pneumoniae* ATCC 13883

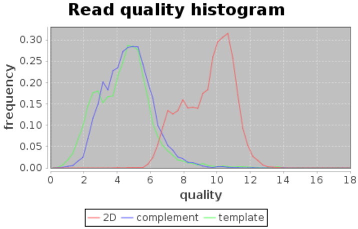

(d) Mixture sample

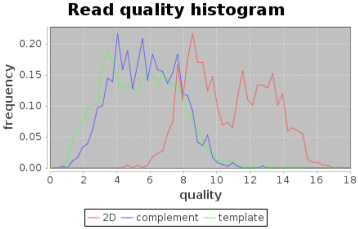

(e) *K. pneumoniae* ATCC BAA-2146

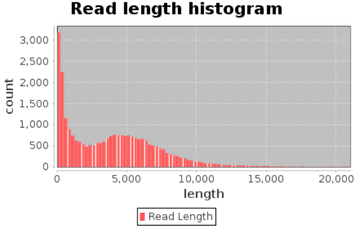

(f) *K. quasipneumoniae* ATCC 700603

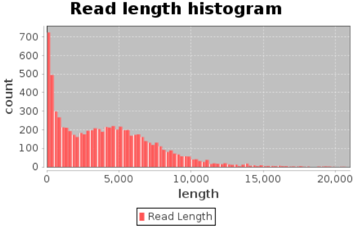

(g) *K. pneumoniae* ATCC 13883

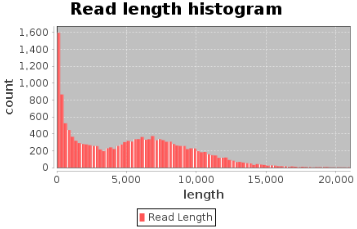

(h) Mixture sample

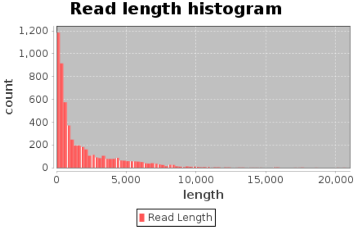

Supplement: Additional file 1 — Figure S1. Histograms of read quality (in Phred score) and read lengths of four MinION sequencing runs. (PDF 112 kb) [file 13742_2016_137_MOESM1_ESM.pdf]
