# Supplementary figures and images for: Streaming algorithms for identification of pathogens and antibiotic resistance potential from real-time MinIONTM sequencing
Source: Gigascience. 2016 Jul 26;5(1):32. doi: 10.1186/s13742-016-0137-2 (PMC4960868; doi:10.1186/s13742-016-0137-2)

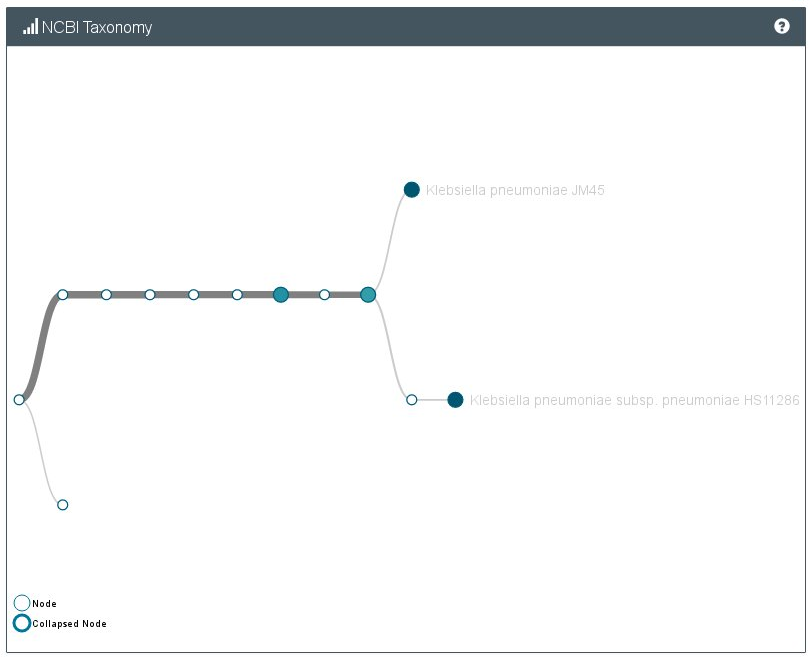

Supplement: Additional file 2 — Figure S2. Screen shot of What’s In My Pot (WIMP) analysis of the clinical sample after three hours of sequencing. (PNG 58 kb) [file 13742_2016_137_MOESM2_ESM.png]
